# The Effect of Degeneration on Internal Strains and the Mechanism of Failure in Human Intervertebral Discs Analyzed Using Digital Volume Correlation (DVC) and Ultra-High Field MRI

**DOI:** 10.3389/fbioe.2020.610907

**Published:** 2021-01-21

**Authors:** Saman Tavana, Spyros D. Masouros, Nicoleta Baxan, Brett A. Freedman, Ulrich N. Hansen, Nicolas Newell

**Affiliations:** ^1^Biomechanics Group, Department of Mechanical Engineering, Imperial College London, London, United Kingdom; ^2^Royal British Legion Centre for Blast Injuries Studies, Department of Bioengineering, Imperial College London, London, United Kingdom; ^3^Biological Imaging Centre, Central Biomedical Services, Imperial College London, Hammersmith Hospital Campus, London, United Kingdom; ^4^Department of Orthopaedic Surgery, Mayo Clinic, Rochester, MN, United States

**Keywords:** disc degeneration, digital volume correlation (DVC), internal 3D strain, vertebral fracture, magnetic resonace imaging (MRI), endplate fracture, intervertebral disc (IVD)

## Abstract

The intervertebral disc (IVD) plays a main role in absorbing and transmitting loads within the spinal column. Degeneration alters the structural integrity of the IVDs and causes pain, especially in the lumbar region. The objective of this study was to investigate non-invasively the effect of degeneration on human 3D lumbar IVD strains (*n* = 8) and the mechanism of spinal failure (*n* = 10) under pure axial compression using digital volume correlation (DVC) and 9.4 Tesla magnetic resonance imaging (MRI). Degenerate IVDs had higher (*p* < 0.05) axial strains (58% higher), maximum 3D compressive strains (43% higher), and maximum 3D shear strains (41% higher), in comparison to the non-degenerate IVDs, particularly in the lateral and posterior annulus. In both degenerate and non-degenerate IVDs, peak tensile and shear strains were observed close to the endplates. Inward bulging of the inner annulus was observed in all degenerate IVDs causing an increase in the AF compressive, tensile, and shear strains at the site of inward bulge, which may predispose it to circumferential tears (delamination). The endplate is the spine's “weak link” in pure axial compression, and the mechanism of human vertebral fracture is associated with disc degeneration. In non-degenerate IVDs the locations of failure were close to the endplate centroid, whereas in degenerate IVDs they were in peripheral regions. These findings advance the state of knowledge on mechanical changes during degeneration of the IVD, which help reduce the risk of injury, optimize treatments, and improve spinal implant designs. Additionally, these new data can be used to validate computational models.

## Introduction

Three in four people will experience low back pain (LBP) at some point in their life and in terms of disability it is ranked first among the 291 conditions that were reviewed in a 2010 Global Burden of Disease study (Hoy et al., 2014). Lumbar intervertebral discs (IVD) have been shown to be a source of chronic LBP (Kuslich et al., 1991), and previous studies have shown a link between IVD degeneration and LBP (Luoma et al., 2000). However, the details of this link are not fully understood, likely due to the complex mechanical environments in both non-degenerate and degenerate IVDs, and lack of a reliable measurement technique to identify stress/strain concentration within whole IVDs. Painful IVDs are commonly structurally disrupted (Freemont et al., 1997), contain inward collapse of inner annulus fibrosus (AF) (Schwarzer et al., 1995), and show abnormal stress concentrations (McNally et al., 1996) that can compress nerve roots and cause pain. Therefore, full-field 3D internal strain measurement within both non-degenerate and degenerate IVDs can advance the state of knowledge on physiological consequences of mechanical changes during degeneration. Degeneration causes severe changes in the mechanical response of IVDs (Adams et al., 1996), particularly in terms of alterations in the distribution of forces through the vertebral bodies (VB) and endplates, consequently affecting the mechanism of spinal fracture (Adams et al., 2006). Vertebral fracture is experienced by 12–20% of people over fifty years old (Melton et al., 1993; O'Neill et al., 1996), and significantly increases mortality rates even in relatively healthy patients (Cauley et al., 2000). Previous studies have shown that the mechanism of vertebral fracture is associated with the quality of the underlying trabecular bone, endplate thickness (Zhao et al., 2009), and endplate deflection (Jackman et al., 2014), however, the influence of 3D IVD strain patterns on failure mechanisms has not previously been investigated. Understanding how the IVD fails is important to implant designers who can adapt designs to avoid high loads being transferred through weak areas of the motion segment. Additionally, understanding the relationship between mechanical behavior of the IVD and degeneration is important for improving treatments for LBP patients, such as implants and tissue engineered IVDs.

Previous studies have investigated the effect of degeneration on the internal mechanics of human IVDs using *in vivo* (Nachemson, 1965) and *in vitro* (Nachemson, 1965; Mcnally and Adams, 1992; Adams et al., 1993, 1996, 2000; Skrzypiec et al., 2007) pressure measurements with thin transducers inserted into the IVD. These studies have shown a shift of high compressive stresses from the nucleus pulposus (NP) to annulus fibrosus (AF), particularly to the posterior AF, with degeneration. IVD deformations and strains in the degenerate and non-degenerate IVDs have also been quantified and compared by inserting wires through specimens and using radiographs to track the movement of these wires under compressive load showing that degeneration increases circumferential and radial strains, particularly in the posterolateral AF (Tsantrizos et al., 2005; Costi et al., 2007; Amin et al., 2019). However, all these studies have relied upon techniques that cause damage to the IVD, that may alter the internal mechanics, and induce residual strains (Bonnevie et al., 2019), thus rendering the technique impractical for clinical applications.

Magnetic resonance imaging (MRI) in combination with image registration is a promising non-invasive technique to quantify the mechanical behavior of human IVDs. Digital image correlation (DIC) in combination with 3T MRI has been used to investigate the effect of degeneration, loading modalities, and nucleotomy on IVD strain distributions in 2D (O'Connell et al., 2007, 2011b,c), and displacement-encoded imaging has been used to measure 2D deformations and strains in non-degenerate IVDs under cyclic load using 3T MRI (Chan and Neu, 2014). However, the 2D nature of these techniques means that they are not sensitive to out-of-plane loads and cannot evaluate 3D strain components. Furthermore, higher resolution strain measurements are achievable using 7T or 9.4T MRI compared with 3T MRI. 3D internal strains have been measured in human IVDs in two previous studies (Yoder et al., 2014; Showalter et al., 2016) using non-rigid registration (Avants et al., 2008) of 7T MRIs, and in rat IVDs using digital volume correlation (DVC) in combination with synchrotron computed tomography (Disney et al., 2019). While, the effect of degeneration on the internal strains and mechanism of vertebral fracture was not evaluated.

This study therefore has two main aims. Firstly, to implement a previously developed technique based on digital volume correlation (DVC) and ultra-high field (9.4T) MRI (Tavana et al., 2020a) to investigate the relationship between 3D IVD strains and degeneration. And secondly, to determine whether there is a link between 3D IVD strain patterns and the mechanism of failure within a spinal motion segment.

## Methods

### Sample Preparation

Ten human lumbar functional spinal units (FSU) were obtained from four male donors (Table 1). Ethical approval was obtained from the Tissue Management Committee of the Imperial College Tissue Bank ethics committee (ethical approval number: 12/WA/0196). Samples were sealed and stored frozen at −20°C but left to thaw overnight in a sealed plastic bag at 4°C for different stages of preparation and testing. Soft tissues were removed with the anterior and posterior longitudinal ligaments kept intact, the posterior elements were removed by cutting through the pedicles at a distance of ~2 mm from the edge of the vertebral body. Each vertebral body was then transversely cut into two halves. MRIs (3T Magnetom Skyra, Siemens; Erlangen, Germany—T2-weighted turbo spin echo sequence, time to repetition (TR) = 12,730 ms, time to echo (TE) = 105 ms, flip angle = 160°, voxel size 1.0 × 1.0 × 5.0 mm) and CTs (IVIS SpectrumCT Imaging System, Caliper Life Sciences, Hopkinton, MA, USA—voxel size 0.15 × 0.15 × 0.15 mm) were acquired of the samples using clinical scanners. The level of degeneration was identified through an average of Pfirrmann grades (2001) adjudged by three observers from the sagittal MRI scans of each sample. For the scope of this study samples with a Pfirrmann grade ≤2 were classed as non-degenerate and samples with a Pfirrmann grade ≥3 were classed as degenerate. Central IVD heights were calculated as an average of measurements made by three observers from the CT scans using Mimics (Materialize HQ, v.19.0, Leuven, Belgium). Samples were then potted with the transverse plane of the disc aligned parallel to the base of the mounting pots of a custom-made, non-magnetic MRI compatible compression rig, and fixed in place using polymethyl methacrylate (PMMA). The MRI compatible rig have been described in more detail in Tavana et al. (2020a), and the potting method to ensure that the mid-plane of each IVD was perpendicular to the axis of loading is described in more detail in Newell et al. (2019).

**Table 1:** Sample details.

**Sample No**	**Age (years)**	**Level**	**Pfirrmann grade**	**Central IVD height (mm)**
1	22	L3-L4	2	12.8 ± 0.3
2		L4-L5	2	12.1 ± 0.3
3	26	L2-L3	2	9.7 ± 0.0
4		L3-L4	2	11.8 ± 0.2
5		L4-L5	2	12.2 ± 0.4
6	58	L3-L4	4	9.7 ± 0.0
7		L4-L5	3	10.9 ± 0.6
8	53	L2-L3	4	9.3 ± 0.3
9		L3-L4	4	9.9 ± 0.2
10		L4-L5	4	9.1 ± 0.2

*Pfirrmann grades are rounded averages of rankings made by three observers. IVD heights ± standard deviation are an average of three measurements at the center of the IVD made from CT scans*.

### Unloaded and Loaded MRI Scans

**Figure 1:**
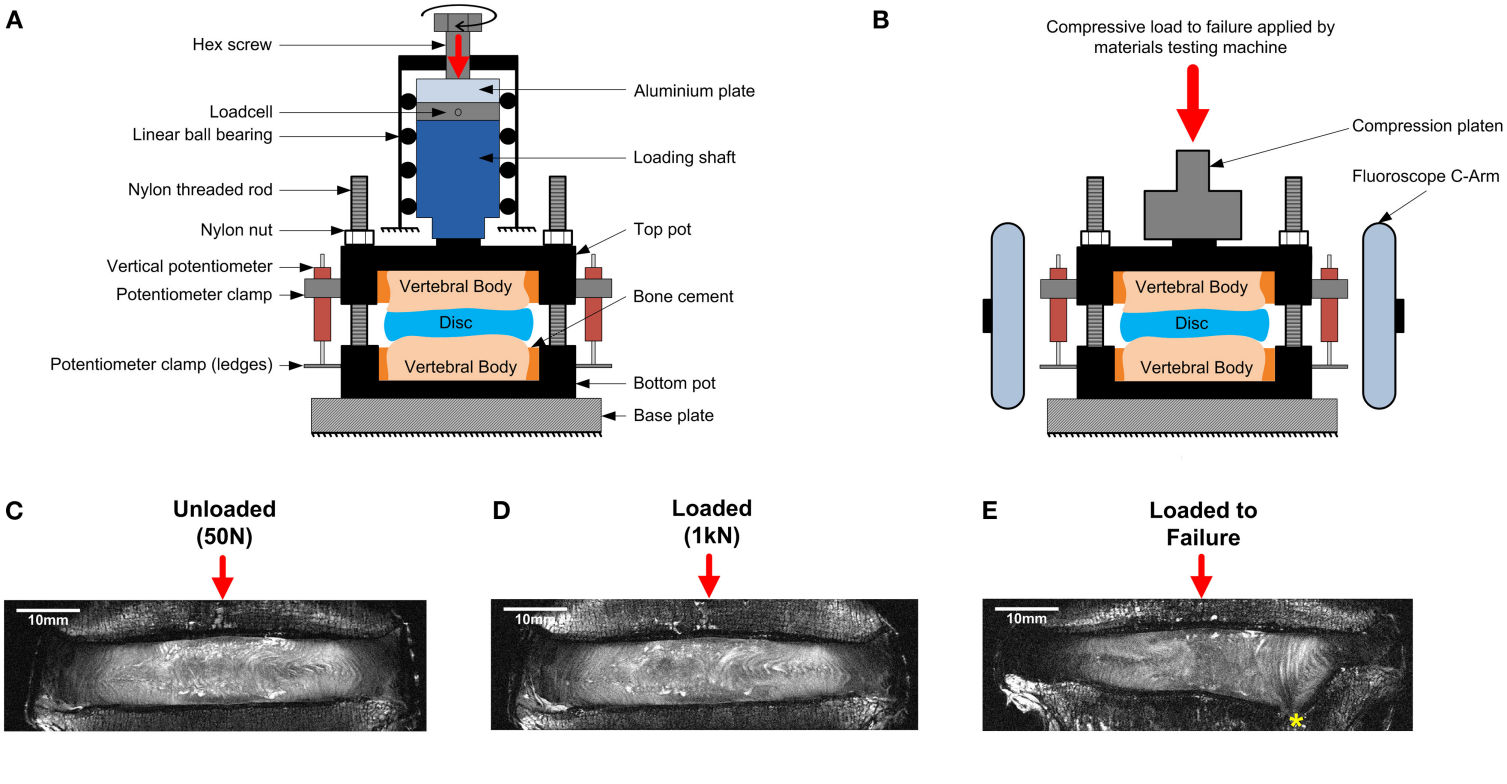
Schematic of **(A)** the MRI compatible loading rig within an external custom-made screw-driven compression rig used to load samples to 50 N and 1 kN, and **(B)** the MRI compatible loading rig within the materials testing machine used for failure tests. The applied loads were purely axial, with no bending or shear movements. A mid-coronal MRI slice of a representative degenerate sample is shown unloaded in **(C)**, loaded to 1kN in **(D)**, and after failure in **(E)**. The endplate fracture location is denoted by an asterisk.

Each sample was imaged using high resolution 9.4T MRI (T_2_-weighted RARE sequence, coronal plane, resolutio*n* = (90 × 90) μm^2^, slice thickness = 800 μm, 17 min scan time, Bruker BioSpec 9.4T, Ettlingen, Germany) in three states; unloaded, axially loaded to 1 kN, and after an axial test to failure. For the unloaded images, each specimen was compressed to a nominal load (50 N) to ensure contact of the loading fixtures using a custom-made, screw driven compression rig (Figure 1A) that was external to the MRI machine. At 50 N, the displacement was recorded using three calibrated potentiometers (S8FLP-10A-1K, Techni Measure Ltd., UK) that were securely attached to the top mounting pot (placed at 120° from each other) and pressed against ledges on the bottom pot. Six nylon nuts attached to threaded rods that passed through the top and bottom pots were then tightened to lock the rig at the displacements recorded by the potentiometers (fixed displacement). The potentiometers were removed so that the sample could be taken out of the external loading rig and placed within the MRI machine for scanning. Specimens were regularly sprayed with phosphate buffered saline (PBS, 0.15 mol/L) to ensure they stayed hydrated during the loading and scanning process.

Following unloaded imaging each sample was removed from the scanner and subjected to 1 kN (loaded) of axial compression using the same method to load the sample to 50 N. The applied load (1 kN ~ 1.2 body weight) corresponds to moderate physiological stress experienced by the IVD during daily activities (Wilke et al., 1999). After loading, the displacement was held (monitored by the potentiometers), and the samples were allowed to relax for 20 min before being imaged using the same MRI sequence. An alignment jig, fitted to the bottom pot using nylon rods and nuts was used to ensure the position of the rig within the MRI scanner was consistent between scans.

### Failure Tests

**Figure 2:**
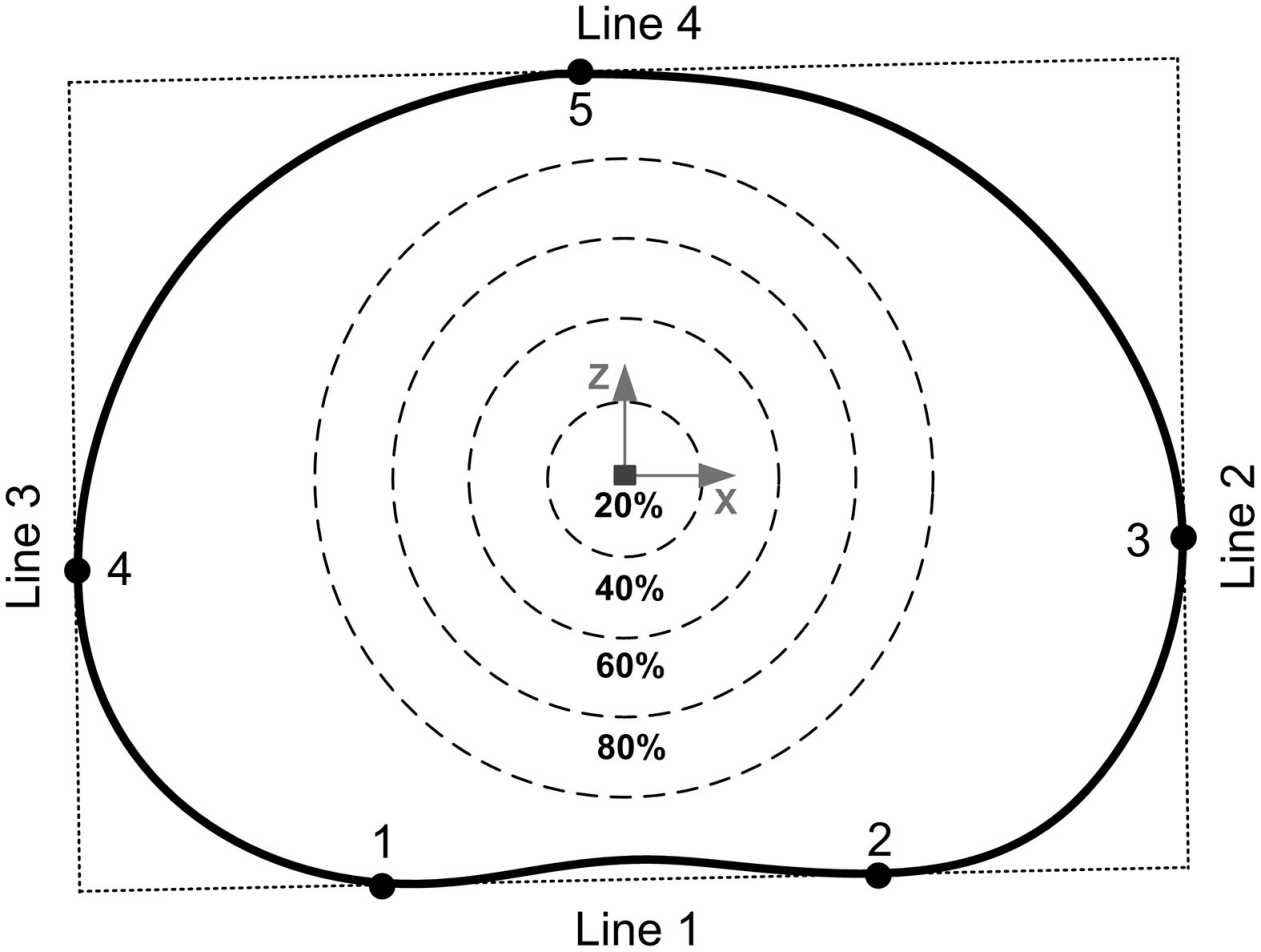
The endplate centroid was located using a method previously developed by Little et al. (2007). Four lines were drawn around the mid-transverse plane of the IVD to create a rectangle: line 1 passed through the two inflections of the posterior annulus boundary (points 1 and 2), lines 2 and 3 were perpendicular to line 1 and passed through the two most lateral points of the annulus boundary (points 3 and 4), and line 4 was perpendicular to lines 2 and 3 passing through the most anterior point of the annulus boundary (point 5). The geometric center of this rectangle was then used as the endplate centroid. The percentages refer to the distance between the furthest of either the most anterior (point 5) or most posterior (points 1 or 2) locations of the IVD in the mid-transverse plane.

After the unloaded and loaded scans samples were unloaded, wrapped in phosphate buffered saline (0.15 m/L), and allowed to recover for 24 h before being tested to failure (O'Connell et al., 2011a). Samples were positioned within a screw-driven materials testing machine (Instron 5866, High Wycombe, UK) and compressed to failure from the unloaded state at a rate of 0.25 mm/s. The point of failure was identified by the first 5% distinct drop of the current force recorded by the loadcell built into the testing machine. This drop indicated a sudden collapse of a load-bearing structure within the sample. Real-time coronal plane fluoroscope images (InsightFD Mini-C-Arm, Fluoroscan, MA) were captured (30 frame/s) of the samples during compression (Figure 1B), such that failure initiation could be identified. Following failure, the test was stopped and nylon nuts were tightened behind the top pot to hold the sample at that level of compression. Samples were scanned for a third time in the 9.4T MRI scanner (Figure 1E), and also carefully dissected after scanning such that the exact location of failure could be identified. For consistency in reporting fracture locations, a local coordinate system was assigned to the center of each endplate. For this, the endplate centroid was identified using a method previously developed by Little et al. (2007), and depicted in more detail in Figure 2. Fracture locations were normalized and mapped onto this template of the IVD's transverse plane. A coordinate system was created on the transverse plane at the endplate centroid where the sagittal plane was defined by a vector passing between the endplate centroid and the posterior junction of the two laminae of the vertebral arch. The positive x direction was to the sample's right, the positive y direction was inferior, and the positive z direction was anterior (Figure 2).

### Image Processing and Digital Volume Correlation (DVC)

The internal 3D deformations were calculated from the unloaded and the loaded scans using DVC as previously described by Tavana et al. (2020a). Briefly, DVC calculates deformations based on dividing the whole volumetric image into small subsets and tracking internal patterns from the unloaded scan (Figure 1C) into the loaded scan (Figure 1D). Before implementing DVC, images were pre-processed using bicubic interpolation between signal intensity values of consecutive slices along the z-direction to transform the raw non-cubic voxels (90 × 90 × 800) μm^3^ to cubic voxels (90 × 90 × 90) μm^3^ (ImageJ 1.49u, National Health Institute, USA). Sample specific 3D binary region-of-interest masks were created for each sample separately by manual segmentation of the IVD using Mimics (Materialize HQ, v.19.0, Leuven, Belgium). These masks were used to exclude surrounding tissues (bone and other soft tissues) from the analysis ensuring that only IVD tissue was analyzed. A combination of the Fast Fourier Transform (FFT) and Direct Correlation (DC) approaches (FFT+DC) with subset size of 56 × 56 × 56 voxels and 50% overlap was used for the DVC analysis using DaVis 8.4.0 (LaVision, Germany). A previous study by this group has shown that FFT+DC is the optimal DVC approach for studies of this kind, that a subset size of 56 voxels (2.52 mm) provides a reasonable compromise between errors and spatial resolution, and that using these settings results in random displacement error below 0.3 voxels (28 μm) and both systematic and random strain errors below 2,280 microstrain (Tavana et al., 2020a). To compare strains in different anatomical regions four areas were manually identified for each specimen using ultra-high field MR images: the anterior AF (AAF), the lateral AF (LAF), the posterior AF (PAF), and the nucleus pulposus (NP).

Additionally, to investigate the effect of inward or outward bulge of the inner AF on the strains in the lateral AF, the left and right lateral volumes of each IVD were analyzed separately (2 from each IVD, with some IVDs having inner bulge on one side and outer bulge on the other) and grouped into either those that had inward bulge (IB) of the inner AF (Group IB) or outward bulge (OB) of the inner AF (Group OB). These two groups were independent of degeneration grade to allow the effect of inward or outward AF bulge to be analyzed without this as a confounding factor.

Due to the large strains that soft tissues exhibit, calculations that incorporate assumptions associated with small strain theories may lead to errors in absolute values, therefore displacement fields calculated by DVC were extracted from DaVis and imported to a custom written MATLAB script (MathWorks, Inc., Natick, MA) to quantify all components of the Green-Lagrange strain tensor using a centered finite differences scheme (Germaneau et al., 2007; Chan et al., 2016; Disney et al., 2018). A Shapiro-Wilk's test was implemented to assess normality in all statistical analyses, and a Levene's test was conducted to determine whether the assumption of homogeneity of variance was legitimate. Differences between non-degenerate and degenerate IVDs were assessed using unpaired *t*-tests (significance level *p* = 0.05, 95% confidence interval). Statistical analysis was performed using SPSS statistics 25 (IBM corp., Armonk, NY). In order to evaluate the effect of sample size on the results of statistical analysis, a measure of effect size (Cohen's d) (Cohen, 1988) and power calculation (Bassani and Galbusera, 2020) were performed using G^*^Power (Heinrich-Heine-Universitat Düsseldorf, Germany) (Faul et al., 2007).

## Results

**Figure 3:**
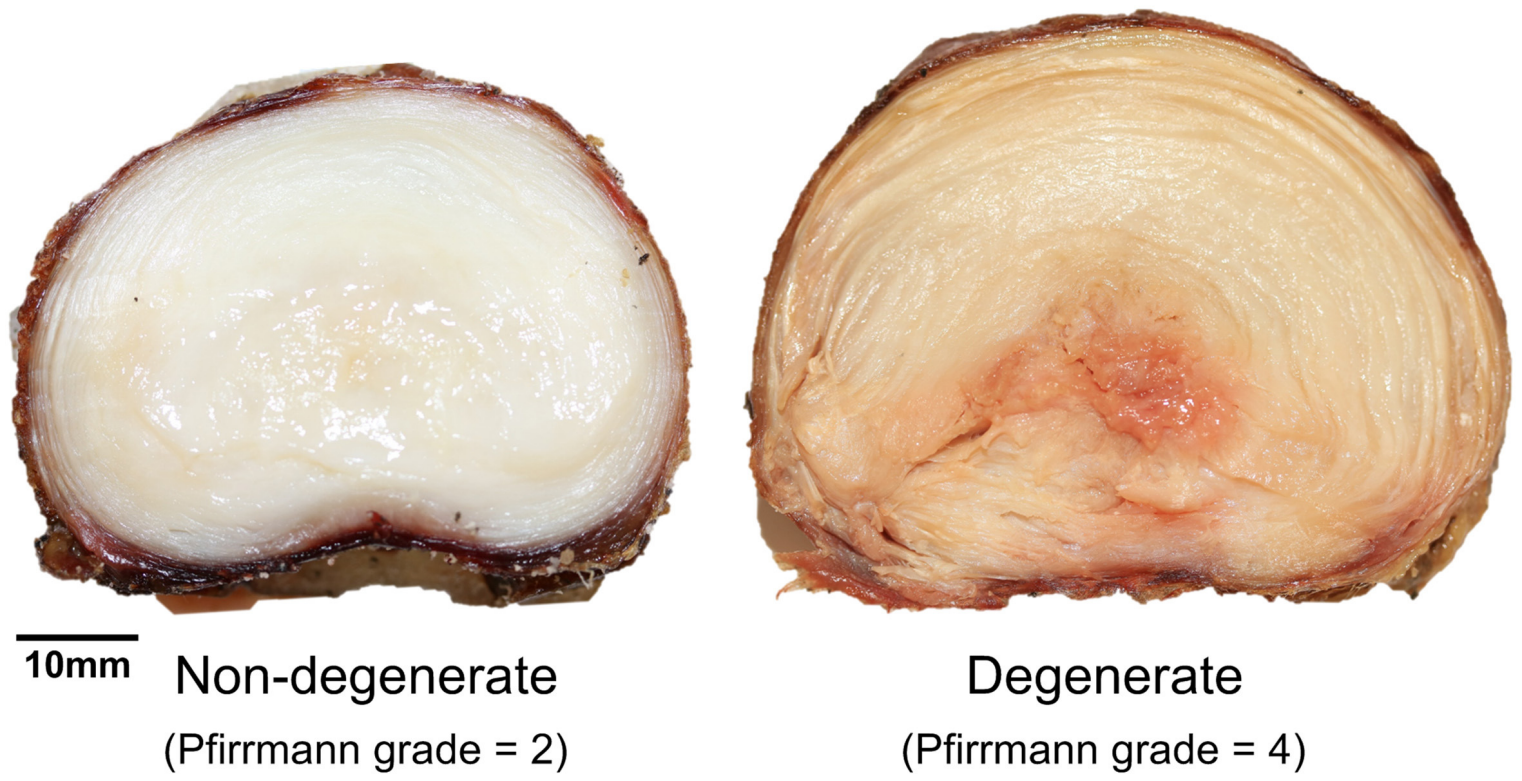
Axial photographs of typical non-degenerate (left, Pfirrmann grade = 2, 26-year-old) and degenerate (right, Pfirrmann grade = 4, 53-year-old) samples taken during specimen dissection after the failure test.

Details of each of the samples are shown in Table 1. No significant differences were observed between failure force (*p* = 0.75) and displacement at failure (*p* = 0.65) of non-degenerate (7.55 ± 1.42 kN, 3.03 ± 0.38 mm, respectively) and degenerate (7.12 ± 1.39 kN, 3.36 ± 1.04 mm, respectively) IVDs. Photographs of a typical degenerate and non-degenerate sample are shown in Figure 3. Analyzing the photographs showed that non-degenerate IVDs were white in color, the NP appeared to be gelatinous, and usually there was no sign of AF tears or delamination. Degenerate IVDs were cream in color, the NP was fibrous, dry and discolored (often red or brown), and annular, radial and circumferential tears were common.

### 3D Strains From the Unloaded and Loaded MRI Scans Using Digital Volume Correlation

#### Effect of Degeneration

Two samples (#3 and #8) were excluded from this part of the study (comparing degenerate and non-degenerate 3D strains) due to an instrumentation failure that meant the magnitude of applied load during the loaded tests was not well-controlled. Axial, minimum 3D principal, maximum 3D principal, and maximum 3D shear strain were obtained for all other samples (**Figure 5**).

**Figure 4:**
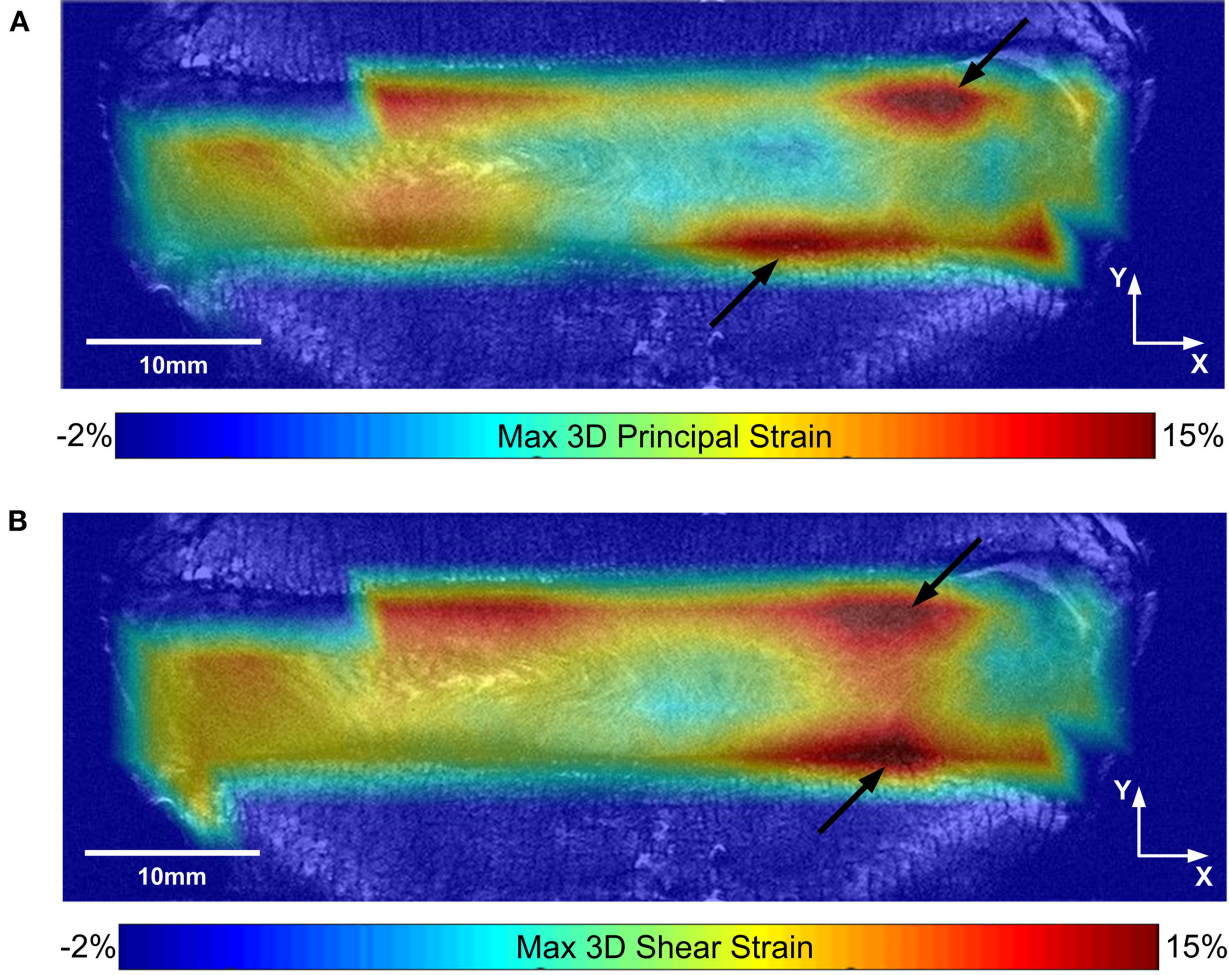
**(A)** Maximum 3D principal (tensile) strain, and **(B)** maximum 3D shear strain maps are shown for a typical sample on the same coronal slice. Black arrows show the location of peak strains close to the endplate.

Analyzing each IVD as a whole, the average axial strain (ε_yy_), average minimum 3D principal strain, and average maximum 3D shear strain in the loaded discs were all significantly larger in the degenerate compared to non-degenerate samples. Specifically, axial strain was −5.37 ± 0.90% in the degenerate vs. −3.39 ± 0.88% in the non-degenerate discs (*p* = 0.03*; d* = *2.23; power 0.87*), minimum 3D principle strain was −9.28 ± 0.76% in the degenerate vs. −6.51 ± 0.56% in the non-degenerate discs (*p* = 0.002*; d* = *4.15; power 0.99*), and maximum 3D shear strains were 8.07 ± 0.65% in the degenerate vs. 5.72 ± 1.02% in the non-degenerate discs (*p* = 0.01*; d* = *2.75; power 0.96*). No significant differences were observed in the maximum 3D principal strain between degenerate (6.87 ± 1.24%) and non-degenerate (4.67 ± 1.39%) groups (*p* = 0.09). In all samples, the site of peak maximum principal (tensile) strain and shear strains were close to the endplates (Figure 4, arrows), with higher values of peak tensile (*p* < 0.001, *n* = 8*; d* = *2.05; power 0.99*) and shear (*p* < 0.007, *n* = 8*; d* = *1.77; power 0.96*) strain at measurement points close (within 2.52 mm) to the endplate (22.5 ± 5.4 and 25.7 ± 4.1%, respectively) compared with points at the mid-transverse plane (13.4 ± 3.2 and 18.7 ± 3.8%, respectively).

**Figure 5:**
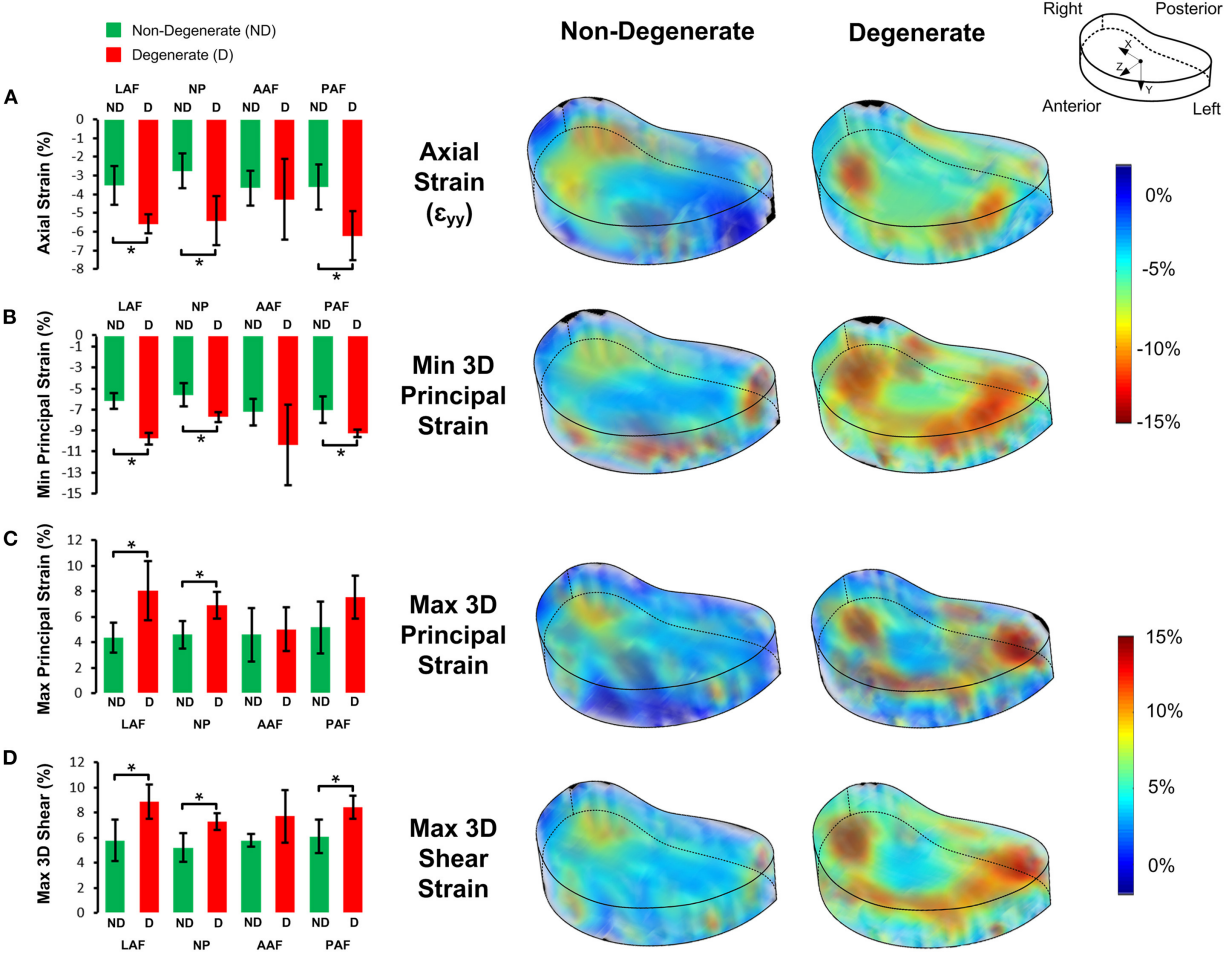
Bar plots of the 3D strain distributions in non-degenerate (ND) and degenerate (D) IVDs in the four anatomical regions (LAF = lateral annulus fibrosus, N*P* = nucleus pulposus, AAF = anterior annulus fibrosus, and PAF = posterior annulus fibrosus) for the **(A)** axial (ε_yy_), **(B)** minimum principal, **(C)** maximum principal, and, **(D)** maximum 3D shear strain components (left). The bars, and error bars represent the average and SD of strain values, respectively and an asterisk (*) denotes significant differences (*p* < 0.05). Transparent views of 3D strain maps for a typical non-degenerate and degenerate IVD are shown on the right.

Figure 5 shows the average strain components in the LAF, AAF, PAF, and NP separately. Significantly higher compressive, tensile, and shear strains were seen in the LAF, PAF, and NP of degenerate, compared to non-degenerate samples (*p* < 0.05). No significant differences were seen in any strain component in the AAF between the degenerate and non-degenerate groups (*p* > 0.05).

### Analysis of Inner Annulus Fibrosus Inward Bulge

**Figure 6:**
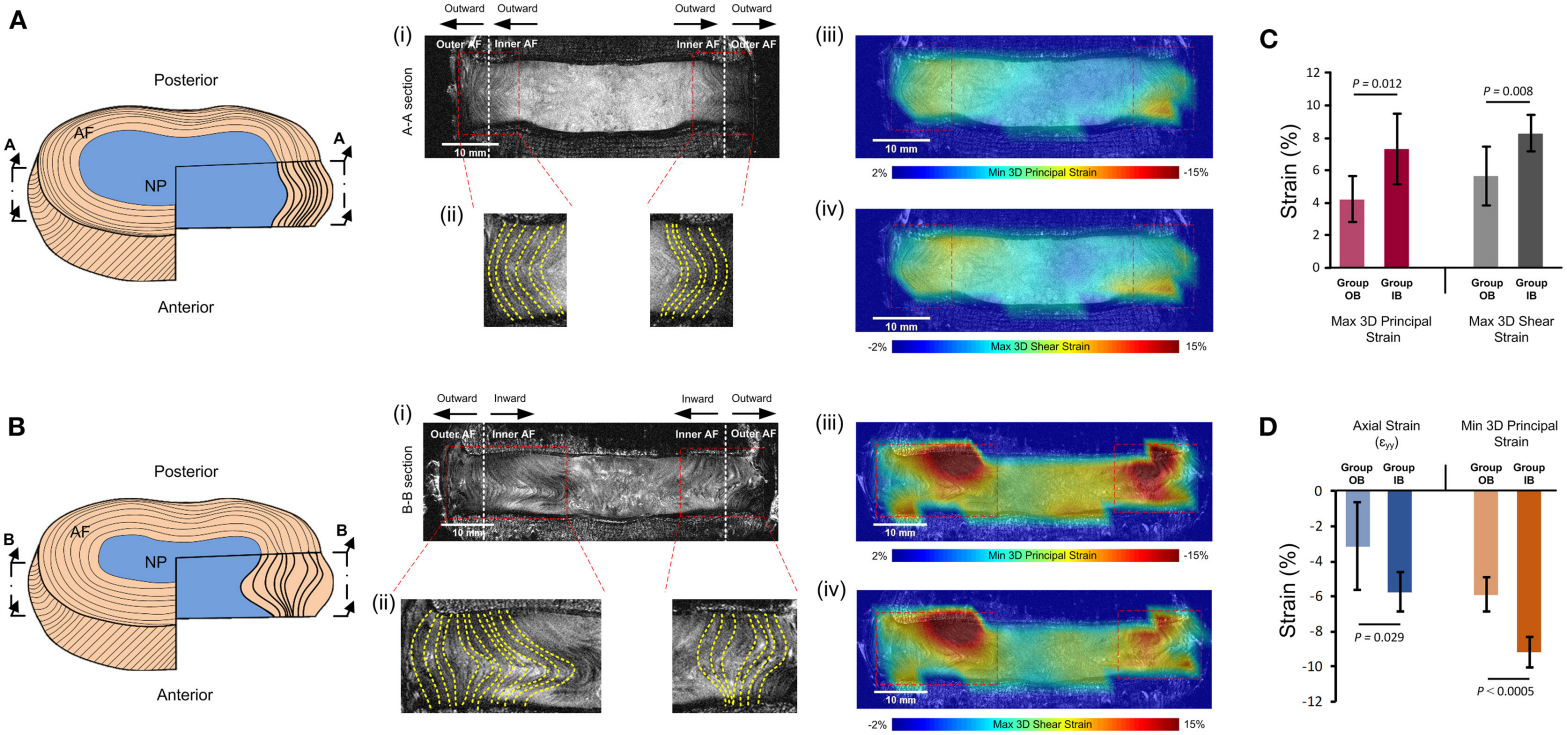
Comparisons of strains seen in specimens with **(A)** outward bulging of the inner AF (Group OB) and **(B)** inward bulging of the inner AF (Group IB). **(A,B)** show schematics of the outward and inward bulge of the inner AF, respectively. **(i)** shows the mid-coronal MRI slice of typical specimens in each group, with **(ii)** showing the left and right lateral regions with yellow dashed lines delineating the morphology of lamellae. The MRI slice, with superimposed minimum 3D principal strains and maximum 3D shear strains are shown in **(iii)** and **(iv)**, respectively. **(C)** compares the 3D principal and 3D shear strains in the two groups and **(D)** compares the axial and minimum principal strains in the two groups. Error bars represent standard deviations.

Of the eight samples (resulting in 16 lateral AF regions), four had inward bulge of the inner AF on both the left and right lateral regions, three had outward bulge of the inner AF on both the left and right lateral regions, and one had inward bulge of the inner AF on one side, but outer bulge of the inner AF on the other. This led to nine lateral AF regions in Group inward bulge (IB) and seven lateral AF regions in Group outward bulge (OB). All lateral AF regions from the degenerate samples were in Group IB and all lateral regions from the non-degenerate samples were in Group OB apart from the left lateral region of one sample where some slight inner AF inward bulge was observed (Figure 6). Generally, inward bulge of the inner annulus into the nucleus led to a significant increase (*p* < 0.05) in the compressive, tensile and shear strain at the site of inward bulge (Figure 6).

### Locations of Failure

**Figure 7:**
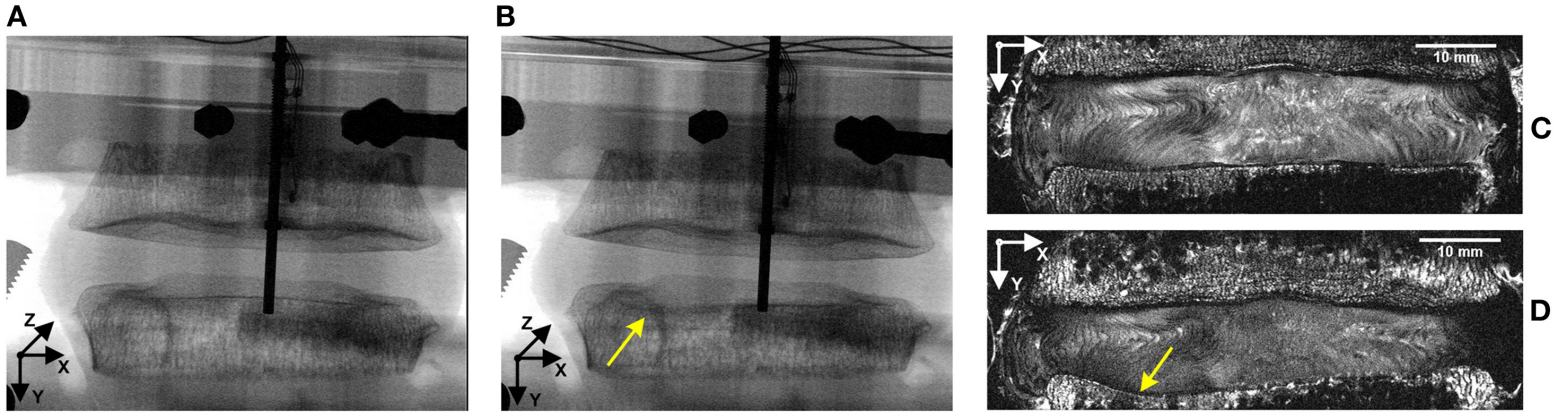
Representative fluoroscope **(A,B)** and MR **(C,D)** images of a sample before **(A,C)** and after **(B,D)** fracture. Combining these two imaging modalities, along with manual dissection of each sample allowed the location of fracture to be identified, with the fluoroscope images being used to identify the initiation of the fracture and the MR images being used to precisely locate the fracture site on a transverse slice. Yellow arrows identify the location of fracture.

**Figure 8:**
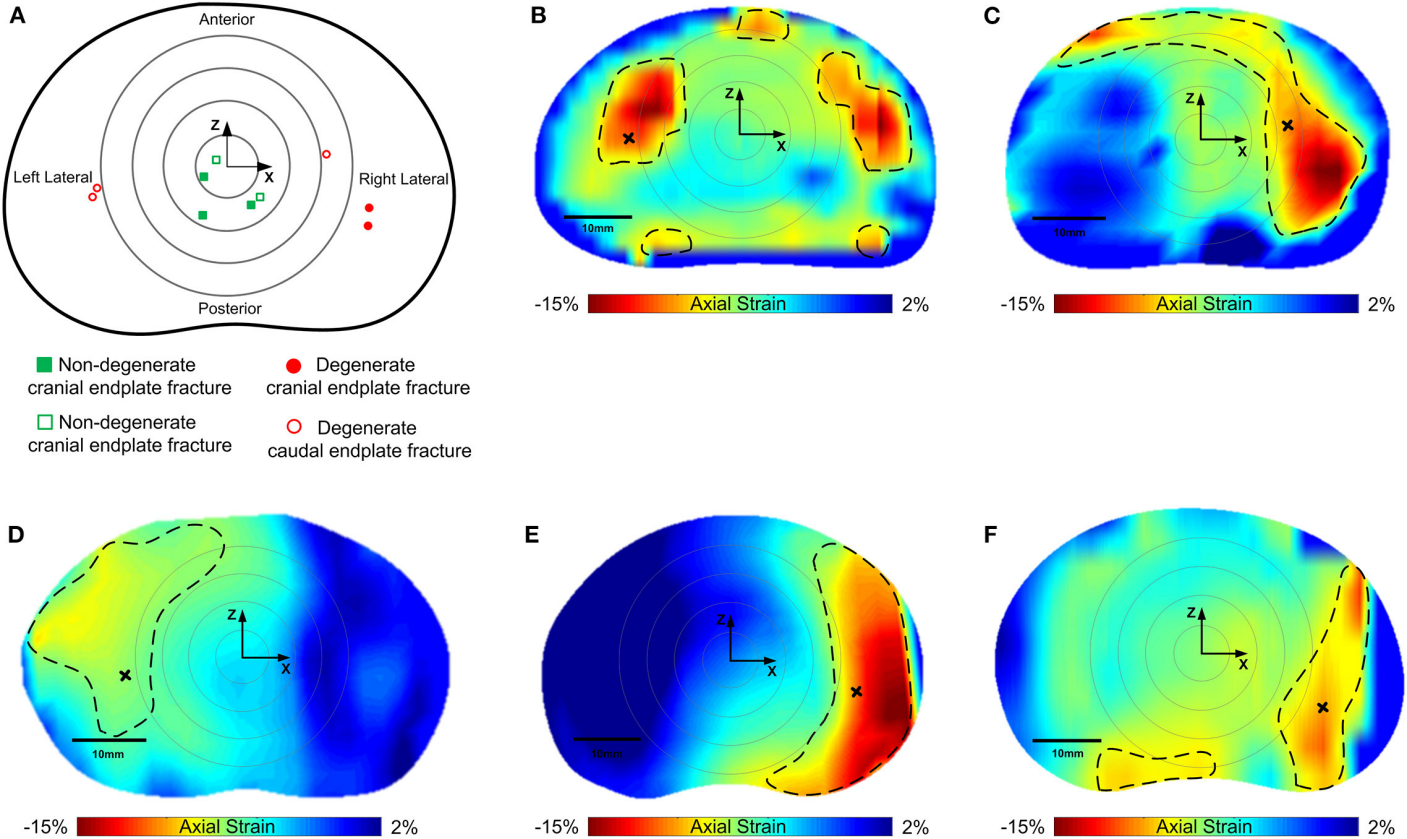
**(A)** Normalized locations of endplate fracture in each of the samples tested. Squares (green) are non-degenerate samples, circles are degenerate (red). Solid points are cranial, and hollow points represent caudal endplate fracture. Concentric rings represent zones 20, 40, 60, and 80% of the distance between the furthest of either the most anterior or the most superior points of the vertebral body in the sagittal plane. **(B–F)** are axial strain distributions in the transverse plane of the degenerate IVDs overlaid by the location of endplate fracture. Non-degenerate discs are not shown as there was no correlation between regions of high axial strains and fracture location. Regions inside of the dashed lines indicate the high axial strain regions, and the areas outside represent low axial strain regions. Black crosses denote locations of fracture in the strain maps.

Compressive loading led to endplate fracture in all samples with half failing through the cranial endplate and half through the caudal. There was no correlation between the side of VB fracture (cranial or caudal) and the grade of IVD degeneration. Coronal views of MR and fluoroscope images from a typical sample before and after fracture are shown in Figure 7, and the normalized locations of endplate fracture are shown in Figure 8A. The location of fracture for all non-degenerate samples were within 40% of the distance between the endplate centroid and the furthest of either the most anterior or most posterior part of the IVD in the transverse plane (see Figure 2). Fracture in the degenerate samples was closer to the outer boundary of the endplates with no fractures seen within 60% of the distance between the endplate centroid and the furthest of either the most anterior or most posterior part of the IVD in the transverse plane, and with four out of five of the samples having fracture locations over 80% of that distance. All fractures in the degenerate samples were either left or right lateral with no fractures seen in the anterior or posterior portions of the endplates. Both the strain pattern analysis of IVDs and morphological analysis of imaging data exhibited high compressive axial strains linked with the locations of endplate fracture in the degenerate IVDs. Combining the strain distributions in the transverse plane of the IVD tissue with the location of endplate fracture (Figures 8B–F) demonstrated that endplate fractures were always located in the high axial strain regions in the degenerate IVDs. High axial strain regions were defined as a group of subsets where the magnitude of measured axial strain was more than 25% higher than the average axial strain on the whole transverse plane from which that subset belonged. This threshold was defined to allow a repeatable, quantitative approach to identifying regions with high strain concentrations. The boundaries of high axial strain regions were identified using a custom written MATLAB script (MathWorks, Inc., Natick, MA) and shown in Figures 8B–F.

## Discussion

In this study, the effect of degeneration on internal human IVD strains and the mechanism of spinal failure was investigated. For the first time, high resolution 3D internal strains were measured throughout the whole IVD non-invasively using DVC in combination with 9.4T MR images under 1kN of compressive axial load.

Degenerate IVDs had higher (*p* < 0.05) 3D axial strains (58% higher), minimum principal 3D strains (43% higher), and maximum 3D shear strains (41% higher), in comparison to the non-degenerate IVDs. The increase in intradiscal 3D compressive (minimum principal strains), tensile (maximum principal strains) and shear strains with degeneration, particularly in the posterolateral AF (Figure 5) supports the findings of previous 2D studies that have shown increased compressive axial and tensile radial strains in degenerate IVDs (Tsantrizos et al., 2005; O'Connell et al., 2011c). As with this study, O'Connell et al. (2011c) subjected IVD samples to 1 kN of axial load. Although they only reported 2D strains at discrete locations, their average axial strains in non-degenerate and degenerate IVDs were similar to the 3D strains reported in this study (3.6 vs. 7.1% for non-degenerate and degenerate IVDs, respectively in O'Connell et al., 2011c compared to 3.4 vs. 5.4% in the non-degenerate and degenerate IVDs in this study). Two recent studies (Yoder et al., 2014; Showalter et al., 2016) have calculated 3D strains within the AF of human degenerate IVDs using 7 T MRI and non-rigid registration. These studies did not investigate the effect of degeneration on strains and loaded specimens to a predetermined axial strain, rather than a load and therefore quantifiable comparisons between studies are not possible but higher axial strain values were also reported in the lateral and posterolateral AF in both these studies as was reported here.

Our findings show that degeneration caused greater changes in axial, 3D minimum principal, and 3D maximum shear strains in the posterior AF than the anterior AF where no significant differences were seen in any strain components between degenerate and non-degenerate samples (Figure 5). This supports the finding of studies that have used pressure transducers to show significantly greater increases in axial stresses in the posterior AF compared to the anterior AF with degeneration (Adams et al., 1993, 1996; Skrzypiec et al., 2007). The full-field 3D strain maps obtained in this study also show that the lateral and posterolateral AF in degenerate IVDs experience significantly higher (*p* < 0.05) compressive, tensile, and shear strains compared to non-degenerate IVDs (Figure 5). The high tensile strain concentration at the posterior and posterolateral AF of degenerate IVDs seen in this study could be the reason that radial tears are common in these regions of degenerate IVDs (Osti et al., 1992). Additionally, the high maximum 3D principal and shear strains seen close to the AF/NP-endplate interface (Figure 4, arrows) could be the cause of endplate related failures such as peripheral rim lesions. The high shear strain observed at the IVD-endplate interface in this study is in line with findings reported in finite element studies (Goel et al., 1995; Eberlein et al., 2001).

The increase in NP axial and minimum 3D principal strains with degeneration (Figures 5A,B) suggests a decrease in NP stiffness. Aging and degeneration results in reduction of NP proteoglycan concentration (Adams and Roughley, 2006), and have a negative effect on metabolic transport (Malandrino et al., 2011, 2015). IVD proteoglycan contains high concentrations of anionic glycosaminoglycan, which provides osmotic properties that retains water, helping to resist compressive loads (Dickson et al., 1967; Jahnke and McDevitt, 1988; Iatridis et al., 1996; Adams and Roughley, 2006). Therefore, a reduction in proteoglycan concentration in the NP is likely to lead to a decrease in NP stiffness. Degenerative changes may be initiated by structural failures such as endplate fractures or AF tears that increase the disc space filled by the NP leading to a depressurisation of up to 57% (Adams et al., 1993). This depressurisation can also lead to cell-mediated changes, for example chondrocytes, that are sensitive to compressive stresses, to produce less proteoglycan (Ohshima et al., 1995; Adams and Roughley, 2006). This can result in a further reduction in the compressive load bearing potential of the NP and therefore inward bulging of the inner AF, as was seen both in this study (Figure 6) and others (Osti et al., 1992; Tanaka et al., 1993; Adams et al., 1996, 2000; Adams, 2004). The inward bulging of the inner AF in degenerate IVDs may have resulted in the higher (*p* < 0.05, Figure 5) compressive, tensile, and shear strains in the lateral AF (Figure 6), which may lead to circumferential tears (delamination) and further degeneration (Goel et al., 1995). As a result, this characteristic of degenerate IVDs should be considered by implant designers, particularly for treatments such as NP replacements, which may benefit from restoring internal pressures that push the inner AF outwards, and computational modelers who would need accurate internal IVD geometries to capture this behavior.

In agreement with previous studies (Adams and Roughley, 2006; Zhao et al., 2009; Fields et al., 2010; Curry et al., 2016), the endplate was found to be the spine's “weak link” in pure axial compression. The results of the failure test show that in degenerate IVDs failure locations were placed in the peripheral regions, whereas in non-degenerate IVDs the locations of failure were close to the endplate centroid (Figure 8A). This is likely explained by shift of loading from the NP to the AF within the IVDs as they degenerate. In the non-degenerate IVDs, the pressurized NP focuses loading on the central parts of the endplates (Kurowski and Kubo, 1986), which is the weakest (Grant et al., 2001) and thinnest (Zhao et al., 2009) part. However, degenerate IVDs with depressurized NPs, experienced high axial and tensile strains, particularly close to the endplate of the AF (Figures 4 and 5), which led to fractures in the peripheral regions of the endplates. The fact that endplate fractures were seen in regions that experienced high axial strains in the degenerate IVDs (Figures 8B–F), makes it reasonable to conclude that IVD degeneration plays an important role in the mechanism of vertebral fracture, and axial strains could be used to identify locations with higher risks of fracture in degenerate IVDs. The effect of disc degeneration could be an important parameter in the management of osteoporotic fractures of vertebral body when performing a vertebroplasty in that the location of cement injection could be optimized to strengthen the region of vertebral body that has a higher risk of fracture.

No significant differences were observed between non-degenerate and degenerate IVDs in terms of displacement or load at failure in this study. Yoganandan et al. (1989) axially compressed human cadaveric functional spinal units and found that loads at failure were significantly higher in non-degenerate than degenerate samples but displacement at failure was similar between the two groups. The failure loads in non-degenerate samples were similar between this study and Yoganandan et al. (1989) (7.12 vs. 9.02kN, respectively), however there was a marked difference in failure loads in the non-degenerate samples (7.55 vs. 4.38kN, respectively). The fact that Yoganandan et al. (1989) tested functional spinal units, rather than just intervertebral discs, and the differences in definitions of IVD degeneration (Pfirrmann grading in this study, and analysis of a transverse section of the IVD following testing in Yoganandan et al., 1989) may be the reasons for these differences in failure load.

This study has a number of limitations that should be acknowledged. Firstly, there were a limited number of samples (*n* = 10 for fracture study and *n* = 8 for strain study) obtained from four male donors. Although a power analysis was performed to ensure the sample size was large enough to support the significant differences presented, generalization and extrapolation regarding differences between non-degenerate and degenerate IVDs warrants further investigation in studies with larger sample sizes. Additionally, samples were only subjected to pure axial compression, which is the major load direction when standing. It is known that the patterns of load distribution, strain concentrations, and mechanism of spinal failure change with different modes of loading (O'Connell et al., 2011c; Curry et al., 2016). Therefore, future studies should extend this analysis to investigate IVD response in combined loading and include flexion / extension, axial rotation, and lateral bending. Care should be taken when comparing absolute strain magnitudes because the loading was applied to the discs manually by tightening a hex screw (Figure 1A). While the operator attempted to load the discs at a constant rate, this manual method means the speed could not be controlled perfectly. However, the changes in strain rate due to changes in loading rate are likely to be small and therefore have a negligible effect on the DVC results presented. Finally, the experimental settings in this study deviates from *in vivo* condition in two ways: (1) vertebral bodies were transversely cut into two halves. Although the cut face of the vertebra was pushed against a rigid pot and fixed in position with PMMA, this could have affected the mode of failure of the endplates (Figure 1A), and (2) posterior elements were removed from samples in this study and therefore all compressive loads were transferred to the IVDs which is not physiological. Absolute strain values reported here may therefore deviate from those experienced *in vivo*. However, these limitations will not detract from the differences observed between degenerate and non-degenerate discs as this limitation applies to both groups.

## Conclusions

In this study we assessed the effects of degeneration on the mechanical behavior of human IVDs, and the mechanism of failure using DVC and high-resolution 9.4T MRI. In summary our findings were that:

1- Degeneration caused an increased internal 3D compressive, tensile, and shear strain in the lateral and posterior AF.
2- Inward bulging of the inner AF was observed in all degenerate IVDs and an increase in the AF compressive, tensile, and shear strains was seen at the site of inward bulge, which may predispose it to circumferential tears (delamination).
3- The endplate was the spine's “weak link” in pure axial compression, and the mechanism of human vertebral fracture was associated with disc degeneration. In non-degenerate IVDs the locations of failure were close to the endplate centroid, whereas in degenerate IVDs they were in peripheral regions.

These findings help to understand better the mechanism of IVD internal disruption and spinal failure, which are useful for designing and evaluating surgical implants and treatments such as nucleus replacement and vertebroplasty. In addition, the novel high-resolution 3D strain maps presented in this study could be used as a valuable source for the validation of computational models. Due to the non-invasive nature of this technique, it has the potential to be implemented in patients (Tavana et al., 2020b), and strains may serve as functional biomechanical markers for a range of clinical applications.

## Data Availability Statement

The raw data supporting the conclusions of this article will be made available by the authors, without undue reservation.

## Ethics Statement

This study was reviewed and approved by Tissue Management Committee of the Imperial College Tissue Bank ethics committee (ethical approval number: 12/WA/0196). Written informed consent for participation was not required for this study in accordance with the national legislation and the institutional requirements.

## Author Contributions

ST, UH, and NN designed the study. ST and NN planned and carried out the experiments. NB conducted the MR images. ST analyzed the data and wrote the draft of the manuscript together with NN. UH, SM, BF, NB, and NN revised the manuscript. All authors contributed to the article and approved the submitted version.

## Conflict of Interest

The authors declare that the research was conducted in the absence of any commercial or financial relationships that could be construed as a potential conflict of interest.
